# Deep proteomic dataset of human liver samples obtained by two-dimensional sample fractionation coupled with tandem mass spectrometry

**DOI:** 10.1016/j.dib.2022.108055

**Published:** 2022-03-12

**Authors:** Nikita E. Vavilov, Ekaterina V. Ilgisonis, Andrey V. Lisitsa, Elena A. Ponomarenko, Tatiana E. Farafonova, Olga V. Tikhonova, Victor G. Zgoda, Alexander I. Archakov

**Affiliations:** Institute of Biomedical Chemistry, Pogodinskaya street 10, bld. 7, Moscow, Russia

**Keywords:** Mass-spectrometry, Proteomics, Shotgun, SRM SIS, Fractionation

## Abstract

The data was acquired from 3 normal human liver tissues by LC-MS methods. The tissue liver samples from male subjects post mortem were obtained from ILSBio LLC (https://bioivt.com/). Liver tissue was frozen in liquid nitrogen, transported and shipped on dry ice. The proteins were extracted and purified followed up by trypsin hydrolysis. The peptide mixture was aliquoted and analyzed by different LC-MS approaches: one-dimensional shotgun LC-MS, two-dimensional LC-MS, two-dimensional SRM SIS (Selected Reaction Monitoring with Stable Isotope-labeled peptide Standards). The Shotgun assay resulted in a qualitative in-depth human liver proteome, and a semi-quantitative iBAQ (intensity-based absolute quantification) value was calculated to show the relative protein content of the sample. Absolute quantitative concentrations of proteins encoded by human chromosome 18 using SRM SIS were obtained.

## Specifications Table

**Table:** 

Subject	Omics: Proteomics
Specific subject area	Normal liver qualitative and semi-quantitative proteome analysis. Absolute quantitative analysis of proteins encoded by the 18th human chromosome in liver cells.
Type of data	Table MaxQuant output files Skyline output files
How the data were acquired	Mass spectrometry Proteins were extracted from liver tissue and hydrolysed by porcine trypsin (Promega); HPLC-MS/MS analysis in data-dependent acquisition (DDA) mode (Thermo Scientific UltiMate 3000 RSLCnano system coupled with Thermo Scientific Q Exactive HF-X hybrid quadrupole-Orbitrap mass spectrometer); protein identification with MaxQuant software (version 1.6.3.4) and manually reviewed protein database of the Homo Sapiens proteome provided by UniProt (Dec 2021). MaxQuant was used to analyse raw shotgun MS results. The same peptide mixtures were used to perform fractionation for subsequent DDA analysis. The samples were fractionated by reverse phase HPLC in alkaline conditions (Agilent 1200 Series HPLC system with fraction collector). Collected fractions were analyzed in DDA mode (Thermo Scientific UltiMate 3000 RSLCnano system coupled with Thermo Scientific Q Exactive HF-X hybrid quadrupole-Orbitrap mass spectrometer); protein identification with MaxQuant software (version 1.6.3.4) and manually reviewed protein database of the Homo Sapiens proteome provided by UniProt (Dec 2021). MaxQuant was used to analyse raw shotgun MS results. The same peptide mixtures were used to perform fractionation for subsequent SRM SIS analysis. Standard isotope-labelled peptides were added to each sample prior to fractionation. The samples were fractionated by reverse phase HPLC in alkaline conditions (Agilent 1200 Series HPLC system with fraction collector). Collected fractions were used to perform SRM SIS (Selected Reaction Monitoring with Stable Isotope-labelled peptide Standards). Stable isotope labelled peptides were synthesized and used as internal standards to detect endogenous target peptides of proteins encoded by the human 18th chromosome. SRM SIS analysis was performed using Triple Quad LC/MS system (Agilent 1290 Infinity HPLC system coupled with Agilent 6495 Triple Quadrupole). Skyline software (version 21.2) was used to analyse raw SRM SIS data.
Data format	Raw Analyzed
Description of data collection	The tissue liver samples from male subjects post mortem were obtained from ILSBio LLC (https://bioivt.com/). Liver tissue was frozen in liquid nitrogen, transported and shipped on dry ice. The tissue was homogenized in lysis buffer, sonicated and digested by porcine trypsin. Every peptide sample was analyzed by Shotgun approach and peptides were fractionated by reversed phase chromatography in alkaline conditions and the collected fractions were analyzed by Shotgun LC-MS and SRM SIS approaches.
Data source location	• Institute of Biomedical Chemistry • City/Town/Region: Moscow • Country: Russia
Data accessibility	Repository name: figshare Data identification number (permanent identifier, i.e. DOI number): https://doi.org/10.6084/m9.figshare.19312256.v2 Direct link to the dataset: https://figshare.com/articles/dataset/Normal_liver_tissue_proteome_analysis/19312256 Repository name: ProteomExchange: Permanent identifier: PXD028510, PXD026997 Direct link to the dataset: https://www.ebi.ac.uk/pride/archive/projects/PXD028510/private https://www.ebi.ac.uk/pride/archive/projects/PXD026997/private

## Value of the Data

• These data show the variability of the human liver proteome in terms of semi-quantitative analysis of liver proteome and absolute quantitative analysis of proteins encoded by the human chromosome 18.
• The data of normal liver relative and absolute protein concentrations may be reused to compare it with diseased state liver proteins concentrations.
• The data might be reused for the development of absolute quantitative analysis for desired liver proteins detected in shotgun analysis or the SRM SIS data may be compared to other normal human tissue data to find similarities and differences.

## 1 Data Description

The dataset represents data from an in-depth proteomic analysis of three human liver samples. The data obtained are the results of mass spectrometric analysis by the Shotgun and targeted Mass Spectrometry (SRM SIS). The unprocessed Shotgun files are stored in ProteomExchange repository under PXD026997 identification number. The MaxQuant generated search results are given as Excel workbook (proteinGroupsLiver.xls) stored at figshare repository. The resulting sheets contain information about identified proteins including calculated quantitative data (iBAQ). Targeted analysis covers the proteins encoded by the 18th human chromosome. The raw SRM data are stored in ProteomExchange repository under PXD028510 identification number. The results were analyzed in Skyline software and absolute concentrations of identified proteins were calculated based on peak area ratio of endogenous peptide to heavy isotope labelled peptide. The absolute concentrations of identified proteins are given in supplementary files as Excel workbook (SRM_Liver.xls) stored at figshare repository. Detailed information about SRM method (retention time, collision energy, cell acceleration voltage and quantifier and qualifier ions) you can find in Excel workbook (Chr18 method completed.xls). The data of peptide calibration based on peak area under the curve is given in Excel workbook (Chr18 peptides calibration.xls).

## 2 Experimental Design, Materials and Methods

### 2.1 Protein extraction and trypsin hydrolysis

The liver samples were stored at −80 °C. The frozen tissue was sliced in sterile conditions. Mean slice weight was 1 g. Then the slice was homogenized using Potter-Elvehjem homogenizer at 4 °C with manual pestle in 1 ml of lysis buffer containing 1% sodium deoxycholate, 15% acetonitrile, 4 mM TCEP (tris 2-carboxiethylphosphine), 10 mM PBS pH 7.4, 147 mM NaCl to extract and denaturate proteins. The homogenized samples were sonicated and then heated at 60 °C for 20 min at thermomixer. One ml of 4M Urea was added after the sample cooling (final concentration of Urea 2M) with following SH-groups alkylation performed at ambient temperature in dark place by adding 50 mM 2-CAA (2-chloracetamide) for 30 min. The resulting solution was centrifuged at 12,000 g to remove cell debris and supernatant was aliquoted and frozen at −80 °C for further usage.

Protein samples were 10 times diluted with 100 mM TEAB (triethylammonium bicarbonate) and digested with porcine trypsin (Promega) 100:1 w/w for 18 h at 37 °C. The digestion was stopped by formic acid at a final concentration of 3%. Samples were centrifuged at 10,000 × g at 4 °C for 10 min. Supernatant was evaporated in a vacuum concentrator and reconstituted in 0.5% formic acid. The subsequent peptide mixture purification was performed on Acclaim μ-Precolumn (0.5 × 3 mm, 5μm particle size, Thermo Scientific) precolumn during Shotgun analysis or on C18- XBridge, Waters (4.6 × 250 mm, 5 µm pore size, Waters, Ireland) during reversed phase fractionation and fraction collection.

### 2.1 Reversed phase fractionation in alkaline conditions

The peptides fractionation was carried out on Agilent 1200 Series HPLC system, which consists of degasser, dual micro flow pump, autosampler, UV-detector, fraction collector, column compartment. One hundred µg of peptides in a volume of 20 µl were loaded onto the C18- XBridge, Waters (4.6 × 250 mm, 5 µm pore size, Waters, Ireland) at a flow rate of 0.75 ml/min for 3 min in an isocratic mode of Mobile Phase A (15 mM ammonia acetate in HPLC grade water, pH 9.0). Then the peptides were eluted with a gradient of Mobile Phase B (80% acetonitrile, 15 mM ammonia acetate in HPLC grade water, pH 9.0) at a flow rate of 0.75 ml/min.

Total run time was 60 min, which included the gradient steps according Table 1.

**Table 1:** Gradient of peptide elution used in chromatographic fractionation method (RP in alkaline conditions).

Time	A%	B%	Flow speed (ml/min)
0	98	2	0.75
3	98	2	0.75
4	88	12	0.75
40	60	40	0.75
42	2	98	0.75
47	2	98	0.75
50	98	2	0.75
60	98	2	0.75

### 2.2 Shotgun analysis

The analysis was carried out on the Thermo Scientific UltiMate 3000 RSLCnano system coupled with Thermo Scientific Q Exactive HF-X hybrid quadrupole-Orbitrap mass spectrometer. At first peptides were loaded on Acclaim μ-Precolumn (0.5 × 3 mm, 5 μm particle size, Thermo Scientific) by loading pump with 10 μl/min flow speed and were isocratically washed for 5 min by 5% acetonitrile, 0.1% formic acid, 0.01% trifluoroacetic acid in HPLC grade H_2_O. Then the peptides were separated on analytical column (Acclaim Pep-Map RSLC inner diameter of 75 μm, Thermo Fisher Scientific, Rockwell, IL, USA) at flow rate of 0.3 μl/min by gradient of 0.1% formic acid HPLC grade H_2_O solution (polar phase) and 80% acetonitrile, 0.1% formic acid in HPLC grade H_2_O solution (non-polar phase). Q-Exactive HF-X operated in positive ionization mode with default charge set as 2+, charge states more then 6+ and 1+ were excluded from the analysis. The range of full-MS scan was 310–1500 m/z with resolving power 120,000 at m/z 200 and acquisition gain control target set as 1E6 with 50 ms maximum injection time. Tandem mass spectra was registered with scan range starting from 150 first fixed mass and dynamically calculated maximum m/z dependent on the precursor ion m/z. The MS/MS resolving power was set as 15,000 at m/z 200 and acquisition gain control target was determined as 1E5 with 150 ms maximum injection time. The precursor ion was isolated to MS/MS scan in narrow isolation window of 2.0 m/z with 0.5 m/z offset. Precursor ions were dynamically excluded for 20 s after MS/MS scan with 2 ppm precision. The maximum number of precursor ions allowed to be fragmented in one duty cycle was 25 (loop count 25) with normalized collision energy of 29. Raw MS data were processed by MaxQuant software (1.6.3.4) with built in Andromeda search engine [1]. Protein sequences of the complete human proteome provided by Uniprot (Dec 2021, 20364 protein sequences) was used for protein identification with Andromeda. Target decoy method was applied to calculate peptide and protein FDR (5% and 1%, correspondently). Carbamidomethylation of cysteines was set as fixed modification and protein N-terminal acetylation as well as oxidation of methionines was set as variable modification for the peptide search. The tolerance for MS/MS spectra was set as 20 ppm and 5 ppm for precursor ion. The relative quantitative analysis was performed by calculating iBAQ values for identified proteins.

### 2.3 Solid phase peptide synthesis (SPPE)

The peptides of choice were obtained using solid-phase peptide synthesis on the Overture (Protein Technologies, USA) or Hamilton Microlab STAR devices according to a published method [2]. Isotope-labelled leucine (13C6, 15N), arginine (13C6, 15N4), lysine (13C6, 15N2), or serine (13C3, 15N1) were used for isotope-labelled peptide synthesis. The concentrations of the synthesized peptides were measured via amino acid analysis with fluorescent signal detection of amino acids following acidic hydrolysis of the peptides.

### 2.4 SRM SIS analysis

SRM SIS analysis was carried out on the Agilent 1290 Infinity HPLC system comprised binary pump, thermostatable autosampler with installed 20 µL-loop, thermostatable column compartment, 6-port valve and binary pump coupled with Agilent 6495 Triple Quadrupole equipped with Jet stream ionization source. The MS system operated in positive ionization mode, source parameters were set as follows: gas temp – 250 °C, gas flow – 14 l/min, nebulizer pressure – 17 psi, sheath gas temp – 280 °C, sheath gas flow – 11 l/min, capillary voltage – 3500 V, nozzle voltage – 450 V. The system operated in dynamic SRM mode. Twenty microliters of each sample (20 µg) were loaded onto Eclipse C18 (2.1 × 50 mm, 1.8 μm particles size, Agilent, Palo Alto, CA, USA) column. The column was constantly heated at 45 °C. Separation of peptides was achived by a linear gradient of the mobile phase A (0.1% formic acid and 0.01% TFA in HPLC grade H_2_O) and the mobile phase B (0.1% formic acid, 0.01% TFA and 80% acetonitrile in HPLC grade H_2_O). The gradient started of 3% of B, increased to 11% for 2 min following increase of B to 40% for the next 48 min at 0.3 mL/min flow rate. The column was washed by 97% of B for 5 min at 0.3 ml/min and equilibrated in the initial gradient conditions (3% of B) for 5 min at 0.3 ml/min before the next run. The transitions for SRM scouting of proteins encoded by the 18th human chromosome had been selected from open source data at SRMAtlas [3]. Precursors and fragment ions were isolated by the first and third quadrupole, respectively, in a narrow ±0.65 u (Unit mode) isolation window and within retention time scheduled detection window. Collision energies and cell acceleration voltages were optimized for each individual transition at the method development stage. The complete duty cycle was estimated to 1500 ms with dynamic dwell time depending on the number of concurrent transitions. Data obtained after SRM SIS analysis were processed using Skyline (v. 3.7.0.113117) software [4]. Briefly, the peptide was considered to be detected in the run if the differences between relative intensities for three transitions of endogenous and isotopically labelled peptides did not exceed 25%, and the transition chromatographic profiles of endogenous peptides were identical to the corresponding transitions of stable isotope-labelled peptides. For each peptide, a calibration curve was built and the limit of detection (LOD) was determined in a pure standard solution. When analysing the data, only those signals of endogenous peptides were accepted that exceeded the level of the minimum signal determined during calibration. If the signal was below the LOD but was reproduced in each repetition, such a signal was accepted.

### 2.5 Experiment design

The tissue liver samples from male subjects post mortem were obtained from ILSBio LLC (https://bioivt.com/). Liver tissue was frozen in liquid nitrogen, transported and shipped on dry ice. The tissue was homogenized in lysis buffer, sonicated and digested by porcine trypsin. Obtained complex peptide mixtures were analyzed by Shotgun MS by Thermo Scientific UltiMate 3000 RSLCnano system coupled with Thermo Scientific Q Exactive HF-X hybrid quadrupole-Orbitrap mass spectrometer (DDA), 1 µg of peptides per injection.

The same peptides were used for fractionation and fraction collection by reversed phase chromatography in alkaline conditions (pH 9.0) by Agilent 1200 Series HPLC system. As a result, 24 peptide fractions obtained from 100 µg of digested liver samples were analyzed by Thermo Scientific UltiMate 3000 RSLCnano system coupled with Thermo Scientific Q Exactive HF-X hybrid quadrupole-Orbitrap mass spectrometer.

Liver sample digested proteins in quantity of 300 µg was used to perform reversed phase fractionation and fraction collection in alkaline conditions (pH 9.0) by Agilent 1200 Series HPLC system. Stable isotope standards were added to samples prior to fractionation in alkaline conditions. Resulted fractions were analyzed by SRM approach by Agilent 1290 Infinity HPLC system coupled with Agilent 6495 Triple Quadrupole.

## Ethics Statements

Patient post mortem normal liver samples were purchased from ILSBio biobanking, Hicksville, NY, U.S.A. Informed consent was obtained from local ethics committees. Tissues had no clinical evidence of any pathology.

## Data Availability

Normal liver tissue proteome analysis (Original data) (figshare). Shotgun analysis (Original data) (ProteomExchange). SRM analysis (Original data) (ProteomExchange).

## CRediT authorship contribution statement

**Nikita E. Vavilov:** Writing – original draft, Investigation, Formal analysis. **Ekaterina V. Ilgisonis:** Methodology, Software, Validation. **Andrey V. Lisitsa:** Supervision, Data curation. **Elena A. Ponomarenko:** Writing – review & editing, Resources. **Tatiana E. Farafonova:** Data curation, Methodology. **Olga V. Tikhonova:** Resources, Software, Methodology. **Victor G. Zgoda:** Writing – review & editing, Project administration, Supervision. **Alexander I. Archakov:** Conceptualization, Funding acquisition, Project administration.

## Declaration of Competing Interest

The authors declare that they have no known competing financial interests or personal relationships that could have appeared to influence the work reported in this paper. The authors declare the following financial interests/personal relationships which may be considered as potential competing interests.

## Appendix Supplementary materials

Image, application 1
